# Single amplification PCR targeting mitochondrial genome more sensitive in diagnosing malaria than nested 18S PCR among returned travelers in Bergen, Norway

**DOI:** 10.1186/1475-2875-11-S1-P116

**Published:** 2012-10-15

**Authors:** Christel Gill Haanshuus, Stein Christian Mohn, Kristine Mørch, Nina Langeland, Bjørn Blomberg, Kurt Hanevik

**Affiliations:** 1 National Centre for Tropical Infectious Diseases, Department of Medicine, Haukeland University hospital, Bergen, Norway; 2 Institute of Medicine, University of Bergen, Norway

## Background

Nested PCR is a commonly used molecular technique diagnosing malaria because of its high sensitivity and specificity. However, it is time consuming, has low cost-efficiency and considerable risk of contamination. Using amplification targets presented in multiple copies, such as rRNA 18S locus, increases sensitivity. Mitochondrial targets with a higher copy number might further increase sensitivity.

## Methods

The sensitivity and specificity of two newly designed *Plasmodium* genus-specific single amplification PCR programs, based on previously published primers targeting the 18S locus [1] and the mitochondrial genome [2], were compared with a widely used nested 18S PCR [3]. Analyses in dilution series made from standardised *P. falciparum* reference material were performed, as well as retrospective analyses in 135 blood samples, previously evaluated by routine microscopy, from a cohort of 132 fever patients with potential imported malaria. In addition, sequencing of the 220 bp mitochondrial PCR products was performed.

## Results

The new single mitochondrial PCR detected dilutions down to 0.5 parasites/µl with 97% sensitivity (29/30 parallels), while the single 18S PCR and nested 18S PCR detected 0.5 p/µl with 93% and 87% sensitivity, respectively. All three assays detected positive DNA as low as 0.05 p/µl, though not consistently so by any method. Among the patient samples, 20.7 % (28/135) were evaluated as malaria positive by microscopy and PCR combined. Both single amplification PCR assays identified malaria genus with 100% accuracy compared to 27 positives detected by the nested 18S PCR reference method. The mitochondrial PCR detected one more positive than the 18S PCR assays, which was also positive by microscopy, and had 100 % sensitivity (28/28). Routine microscopy missed two infections detected by all three PCR assays. Sequencing of the genus-specific mitochondrial PCR products revealed different single nucleotide polymorphisms which allowed a species-specific identification of the 28 sequences with following distribution of species; 20 *P. falciparum*, six *P. vivax*, one *P. ovale* and one *P. malariae.*

## Conclusions

Design of PCR programs with suitable parameters and optimization resulted in simpler and faster single amplification assays without reducing the sensitivity and specificity compared to a nested PCR reference method. The new mitochondrial PCR, where the amplification target has a higher copy number than for 18S targets, had in this study highest sensitivity, both among standardised reference and patient material, compared to the 18S PCR assays. Sequencing of genus-specific mitochondrial PCR products might be useful as a species determination method, and merits further investigation.
